# Usability Testing of a New Digital Integrated Health Ecosystem (PainRELife) for the Clinical Management of Chronic Pain in Patients With Early Breast Cancer: Protocol for a Pilot Study

**DOI:** 10.2196/41216

**Published:** 2023-05-12

**Authors:** Marianna Masiero, Chiara Filipponi, Silvia Francesca Maria Pizzoli, Elisabetta Munzone, Luca Guido, Vittorio Andrea Guardamagna, Sara Marceglia, Annamaria Caruso, Roberto Prandin, Marco Prenassi, Vania Manzelli, Chiara Savino, Costanza Conti, Federica Rizzi, Alice Casalino, Giulia Candiani, Francesca Memini, Luca Chiveri, Andrea Luigi Vitali, Massimo Corbo, Alessandra Milani, Roberto Grasso, Silvia Traversoni, Elisa Fragale, Florence Didier, Gabriella Pravettoni

**Affiliations:** 1 Department of Oncology and Hemato-oncology University of Milan Milan Italy; 2 Applied Research Division for Cognitive and Psychological Science European Institute of Oncology IRCCS Milan Italy; 3 Division of Medical Senology European Institute of Oncology IRCCS Milan Italy; 4 Division of Palliative Care and Pain Therapy European Institute of Oncology IRCCS Milan Italy; 5 Università degli Studi di Trieste Trieste Italy; 6 Nuvyta SRL Cologno Monzese Italy; 7 IMS- Istituto di Management Sanitario Bresso Italy; 8 Agenzia di Comunicazione Scientifica Zadig srl Società Benefit Milan Italy; 9 Casa di Cura del Policlinico (CCP) Milan Italy; 10 School of Nursing European Institute of Oncology IRCCS Milan Italy; 11 Department of Biomedicine and Prevention University of Rome Tor Vergata Rome Italy

**Keywords:** decision-making, decision aid, chronic pain, eHealth, patient electronic health record, clinical, technology, mobile application, pilot study, breast cancer, chronic, patient, cancer, pain

## Abstract

**Background:**

Chronic pain (CP) and its management are critical issues in the care pathway of patients with breast cancer. Considering the complexity of CP experience in cancer, the international scientific community has advocated identifying cutting-edge approaches for CP management. Recent advances in the field of health technology enable the adoption of a novel approach to care management by developing integrated ecosystems and mobile health apps.

**Objective:**

The primary end point of this pilot study is to evaluate patients’ usability experience at 3 months of a new digital and integrated technological ecosystem, PainRELife, for CP in a sample of patients with breast cancer. The PainRELife ecosystem is composed of 3 main technological assets integrated into a single digital ecosystem: Fast Healthcare Interoperability Resources–based cloud platform (*Nu* platform) that enables care pathway definition and data collection; a big data infrastructure connected to the Fast Healthcare Interoperability Resources server that analyzes data and implements dynamic dashboards for aggregate data visualization; and an ecosystem of personalized applications for patient-reported outcomes collection, digital delivery of interventions and tailored information, and decision support of patients and caregivers (PainRELife app).

**Methods:**

This is an observational, prospective pilot study. Twenty patients with early breast cancer and chronic pain will be enrolled at the European Institute of Oncology at the Division of Medical Senology and the Division of Pain Therapy and Palliative Care. Each patient will use the PainRELife mobile app for 3 months, during which data extracted from the questionnaires will be sent to the *Nu Platform* that health care professionals will manage. This pilot study is nested in a large-scale project named “PainRELife,” which aims to develop a cloud technology platform to interoperate with institutional systems and patients' devices to collect integrated health care data. The study received approval from the Ethical Committee of the European Cancer Institute in December 2021 (number R1597/21-IEO 1701).

**Results:**

The recruitment process started in May 2022 and ended in October 2022.

**Conclusions:**

The new integrated technological ecosystems might be considered an encouraging *affordance* to enhance a patient-centered approach to managing patients with cancer. This pilot study will inform about which features the health technological ecosystems should have to be used by cancer patients to manage CP.

**International Registered Report Identifier (IRRID):**

DERR1-10.2196/41216

## Introduction

### Chronic Pain in Patients With Cancer

Chronic pain (CP) and its management are critical issues in the care pathway of patients with breast cancer. Accruing evidence stated that 30% of breast cancer survivors reported CP, which is likely to persist up to 10 years after the end of cancer-specific treatments [1-3]. CP might rise throughout the care pathway due to surgical procedures and chemotherapy-induced toxicities. In particular, in the population of individuals with breast cancer, different causes of pain incidence have been identified: 25%-60% related to postsurgical pain; 47% pertaining to adjuvant hormonal treatments (including aromatase inhibitors); and finally, 58% related to long-term chemotherapy-induced toxicities [4]. The experience of CP interferes with psycho-emotional well-being (eg, increasing anxiety, depression, and fatigue), psychological adaptation to the disease, personal relationships (eg, partners, family members, and colleagues), and then return to work [1,5]. Furthermore, when CP is not promptly recognized, it may alter patients’ health-related quality of life, thereby impacting health status, disease, and medication adherence [1,6,7]. Compared to those without CP, patients with breast cancer experiencing CP showed low adherence to anticancer treatments (eg, hormone therapy), with a demonstrated reduction in treatment effectiveness and a 5-year survival rate [1].

Furthermore, compared to hospitalized patients, out-of-hospital patients with cancer have lower pain relief [8]. In particular, patients who live in regions with poor medical resources, an inadequate health system, or low health literacy have an increased risk of experiencing high levels of pain and related disabilities [9]. Besides, this difference in pain management is produced by the fragmented management of cancer survivors [10], causing nonadherence to treatments and follow-ups, poor control of the side effects, and long-term physical and psychological consequences. Thus, the international scientific community has advocated the urgency to introduce interventions that can favor a timely identification of cancer-related CP, which could guarantee continued and tailored management.

### Health Integrated Ecosystems for Cancer Chronic Pain Management

Recent advancements in the field of information and communication technology enable the adoption of a novel and challenging approach to care management in the oncological domain throughout the development of integrated ecosystems [11,12]. A growing and consistent series of studies assert that new integrated health technologies have the potential to improve CP management in patients with cancer and overcome current limitations [12-16]. Furthermore, studies suggested that health technology might well manage physical and psychological morbidities associated with pain experience by allowing continuous monitoring [15] of clinical health status in real time and transmitting these data to their health care providers, incorporating them into patients’ electronic health records [15,17,18].

Among existing supportive tools for CP, mobile health apps have been increasingly applied [13,14] and they help patients with cancer to monitor and reduce pain experience by promoting self-management skills and improving their quality of life [8,14]. Furthermore, mobile apps, if interconnected with the health technology ecosystem and managed by health care providers, increase the patient’s involvement in their care pathway [12]. Overall, this type of technology architecture might achieve different clinical outcomes. First, it monitors the physical and psychological well-being of the patients through self-report questionnaires and momentary ecological assessments (eg, using e-diary), in particular, regular administration of standardized self-report questionnaires and momentary ecological assessments, such as e-diary, effectively track variation in physical and psychological symptomatology, allowing an evaluation of affective instability in CP [15,19]. Second, it favors bidirectional and well-timed communication between health care professionals and patients [7,16], for example, patient-reported outcome measures can be managed by mobile health apps and stored in patients’ electronic health records, favoring a fast and direct clinical response to alarm conditions [20]. Third, it permits scheduled clinical follow-ups, reducing the risk of failing continued clinical monitoring during, for example, the survivorship phase [12]. The strategy is meant to improve the quality of care for both outpatients and inpatients. This latter statement is coherent with the National Cancer Institute declaration [21] that has remarked on the importance of building a strong and consolidated connection between inpatients and outpatients, reducing the risk that cancer survivors, after the end of active treatment, move out from the care system. Fourth, it boosts patients to be more involved in their treatment decisions, permitting them to achieve a shared decision about their care pathway throughout the implementation of decision aids. According to the American Society of Clinical Oncology international guidelines [21] for pain management, patients with breast cancer can choose between pharmacological and nonpharmacological treatments. Thus, patients with breast cancer experiencing CP have to face a series of preference-sensitive decisions related to the treatments and their consequences in the short, medium, and long term [22,23]. Accruing evidence [24,25] has stressed the critical role of decision aids as an excellent strategy to increase the probability of getting a shared and involved decision. Decision aids are tailored tools designed to deliver evidence-based information on the disease, available treatments, and associated risks; additionally, they may help patients think about the treatment alternatives according to a personal outlook [24,25]. Overall, health technologies are recognized as facilitators of shared decision-making (SDM), reducing decisional conflict and enhancing patient satisfaction.

Implementing SDM models improves patients’ knowledge and consolidates the comprehension of their treatment preferences [24,26]. Evidence highlighted that, when implemented on mobile apps, decision aids may support patients by raising awareness of treatment preferences, reducing decisional conflict, and improving adherence to medical treatments [27-30] and SDM [31-33]. We argue that the implementation of the decision aid in clinical practice might be beneficial for patients with early breast cancer in the initial stages of the clinical pathway, that is, immediately after the surgery, when the patients have to face a series of preference-sensitive decisions related not only to the anticancer treatments but also to the management of the pain.

### PainRELife Integrated Big Data Ecosystem and Usability Approach

Despite the importance of health technologies in CP management, only some studies have provided integrated technological solutions supporting health care providers in patient management [20]. In addition, available studies have several methodological limitations (eg, small sample size and short follow-up period). Therefore, additional studies are often needed to evaluate the feasibility and usability of such health technology solutions in the clinical practice for supporting patients with CP throughout the care pathway. The evaluation of the user's usability experience is a fundamental prerequisite to increasing the success rate in implementing technologies into routine clinical practice [34] and gives reliable and quantitative information on the quality of e-tools. While the development of new mobile apps should rely on usability testing and patient-centered design process that integrates patients’ needs [34], before testing for effectiveness and improving adoption and compliance [35].

This pilot study offers an opportunity to test the patients’ usability experience of an integrated technological ecosystem for CP management (PainRELife) in a sample of patients with early breast cancer. Once the usability and effectiveness of PainRELife will be proven, this ecosystem might fill the gap between hospital and home care settings, collecting ongoing, and integrated patient-centered data with the 3-fold future aim to (1) carry out ecological monitoring of the physical and psychological pain experience of the patients; (2) obtain psychoeducational information on pain and available treatments; (3) explore of patients’ preferences and support in SDM on pain treatment options between the physicians and the patients.

## Methods

### Primary End Point

The primary end point of this pilot study is the evaluation of patients’ usability experience after 3 months of using a new digital and integrated technological ecosystem (engagement, judgment on usability, aesthetics, quality of given information, personal perception, and perceived impact on behaviors; *Nu* Platform connected with the PainRELife app) for patients with early breast cancer experiencing CP.

### Secondary End Points

Secondary end points concern the following aspects: (1) assessing the usability of the mobile app in terms of the number of access and usage time; (2) assessing changes in self-efficacy related to CP; and (3) assessing SDM processes between patients and health care professionals.

### Study Design

**Figure 1:**
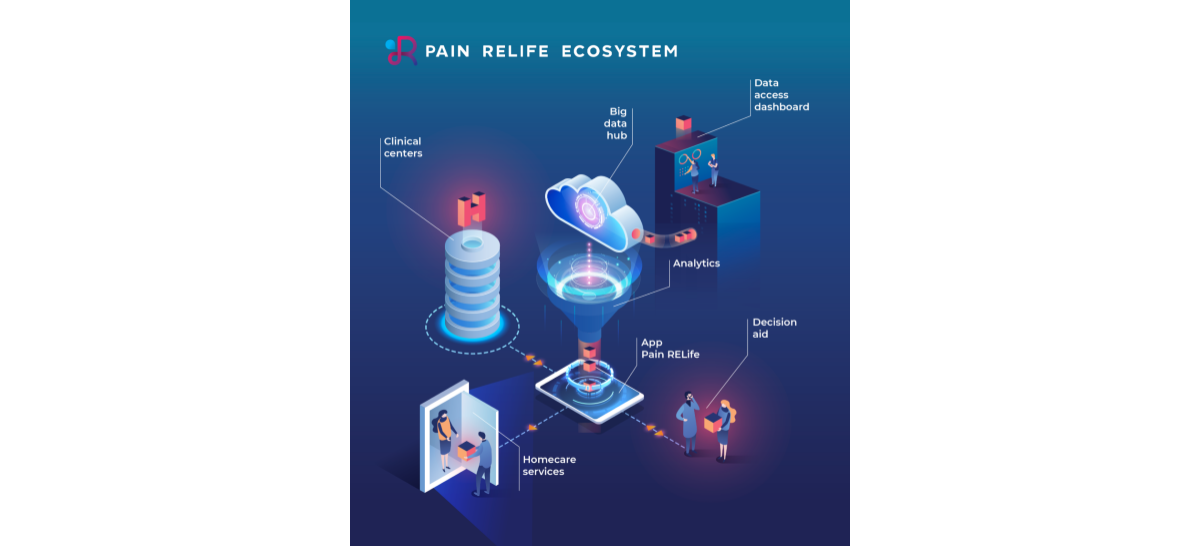
Elements involved in the PainRELife ecosystem and their interconnections.

This is an observational, prospective pilot study nested in a large-scale national project named “PainRELife*, Sustainable and integrated big data ecosystem for continuity of care and decision support for patients with pain*“ (ID 1173269). The PainRELife project aims to develop a cloud technology platform (”Big data HUB“) that is able to interoperate with institutional systems (eg, national electronic health records) and patients’ devices to collect integrated health care data (Figure 1).

### PainRELife Integrated Big Data Ecosystem

The PainRELife is composed of 3 main technological assets integrated into a single digital ecosystem (Figure 1) that were developed during the first year of the project through a collaboration between technical partners and clinical aspects (January 2020 to June 2021).

1. A Fast Healthcare Interoperability Resources (FHIR)–based cloud platform (*Nu* platform) that enables care pathway definition and data collection. The digitized care pathways managed through the *Nu* platform were developed following a consensus procedure that included both the available guidelines and recommendations on CP management and the local experience at the referral centers involved in the project. The digitalized care pathways for pain management became accessible to health care professionals for the data collection on pain, psychological well-being, and decision preferences about treatment options. In particular, the *Nu* platform is the intuitive interface that will be used by health care providers and will permit them to collect and store clinical data (eg, diagnosis, comorbidities, treatments, drugs, pain, anxiety, depression, and emotional distress) and an ongoing monitoring of the patient's health status (from the diagnosis to the follow-ups).
2. A big data infrastructure connected to the FHIR server that analyzes data and implements dynamic dashboards for aggregate data visualization aimed to provide a population-based view for researchers and governance. The dashboards were designed to provide an at-a-glance overview of the population tendencies with respect to the different measures and monitoring procedures. Since these dashboards are not designed for patients, they were not evaluated in this protocol.
3. An ecosystem of personalized apps for patient-reported outcomes collection, digital delivery of interventions and tailored information, and decision support for patients and caregivers (PainRELife app).

The integration of these 3 aspects and the involvement of relevant stakeholders within the CP management process allows the definition of PainRELife as an ”integrated ecosystem.“

### Key Features of PainRELife Mobile App

The PainRELife app was developed to interoperate with the *Nu* platform through the FHIR standard*,* establishing a bidirectional communication channel between health care providers and patients. The PainRELife app has been designed following 3 different steps. First, an extensive and qualitative literature analysis has been performed to define the main issues for patients with breast cancer experiencing CP [36]. In addition, the impact of CP on psychological functioning has been analyzed as the role of the caregivers in the management of the CP experience and how CP impacts different dimensions of the quality of life (sexuality, employment, and returning to work, and psycho-emotional and social determinants). Furthermore, unmet needs in managing CP were evaluated considering multilevel determinants such as sociodemographic, psychological, social, and clinical variables [36]. Finally, the role of technologies in the health care system has been explored to understand the most important aspects that should be considered in the design and development of health technologies. Based on this qualitative analysis, a set of preliminary contents and mock-ups of the mobile app have been developed.

Second, a series of semistructured interviews have been conducted with female patients with breast cancer (N=6) to implement a user-centered design process of the CP mobile app. The semistructured interviews had 3 main goals: evaluating the perceived use of technological support for pain management and the current experience of the patients with the health technologies; evaluating possible interface, contents, functionality, and quality of the mobile app; evaluating a preuser experience of the mobile app through an ad hoc storyboard. Overall, the storyboard is a method used to provide a brief overview of a new mobile app. The storyboard illustrates the main sections of the mobile app and explains the circumstances in which the mobile app should be used.

Finally, all contents of the mobile app have been revised and discussed by an expert group. The group composed of oncologists, palliative physicians, psychologists, and patients. The obtained suggestions have been integrated into the PainRELife app's last version.

**Figure 2:**
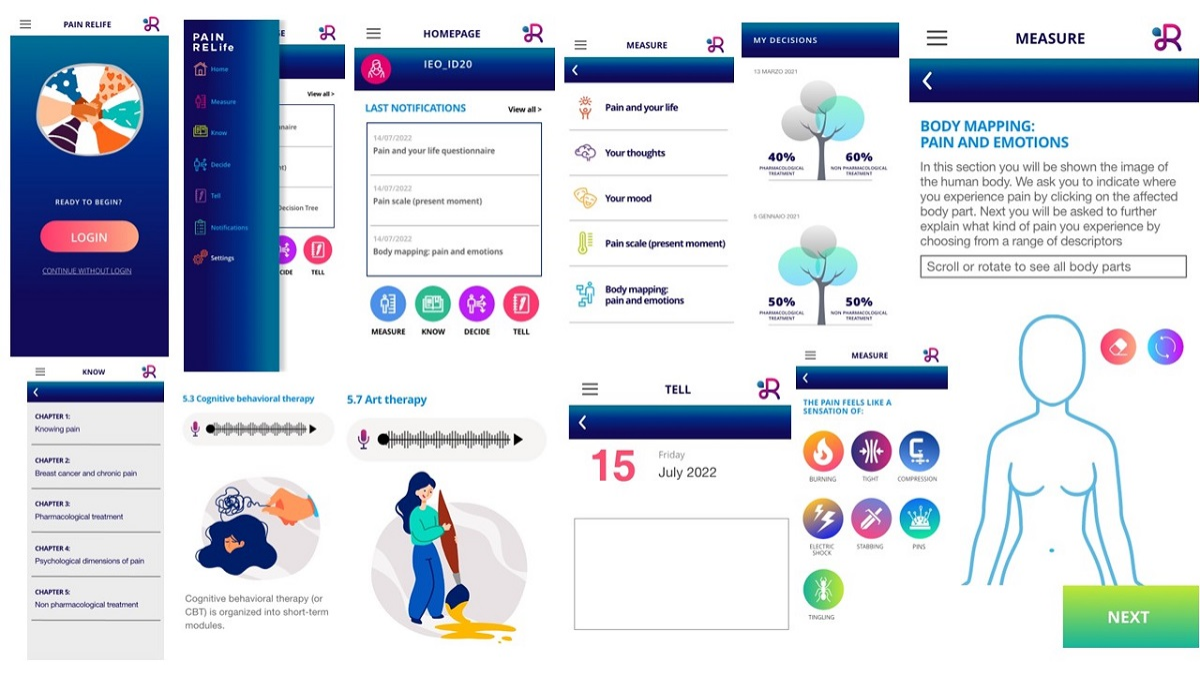
English version of the application PainRELife.

The PainRELife app has been organized into 3 main sections (Figure 2):

1. *Educational Section*: In this section, patients gather evidence-based information on CP and breast cancer. This section aims to improve health literacy using evidence-based information about CP experience during the care pathway (from diagnosis to survivorship). This section contains information on a wide array of topics related to breast cancer diagnosis, cancer pain experience, treatments for CP (pharmacological and nonpharmacological), associated benefits and side effects, and psychological comorbidities. The contents of this section are organized as text, video, and audio files in order to increase the usability rate within different groups of patients with breast cancer.
2. *Pain and Psychological Wellbeing Assessment Section*: It contains a set of self-reported measures evaluating psychological well-being (Hospital Anxiety and Depression Scale; Pain Catastrophizing Scale) [37,38] and pain experience (Brief pain inventory; Visual Analogue Scale [VAS]) [39,40]. This section also includes an e-diary and pain and emotions body mapping exercise. In addition, the e-diary aims to monitor the emotional, cognitive, behavioral, and physical aspects of the CP experience. At the same time, the pain and emotion body mapping exercise request the patients to localize pain and emotions associated using a digital representation of the body (Figure 2). The self-reported measures were implemented with a specific interval time for a total of 3 months with automatic push notifications: Hospital Anxiety and Depression Scale and Pain Catastrophizing Scale monthly; VAS, pain and emotions body mapping exercise, and e-diary each week; and Brief pain inventory 2 times a month. The aim of this section is to (1) permit ongoing monitoring of the patients by health care providers when they are at home and during the follow-up phase and (2) improve the patients' awareness about their own emotional and physical well-being through specific self-monitoring tasks. Overall, the patients will fill questionnaires in the PainRELife app for 3 months. PainRELife app will send an alert to recall the questionnaires.
3. *Decision Aid Section*: It is one of the app's key features (Multimedia Appendix 1). The decision aid comprises 2 parts—profiling patients’ preferences and decision tree for CP treatments. In the first part, each patient fills a set of visual analog scales (ranging from 0 to 100) evaluating the general features of the pharmacological and nonpharmacological treatments. The VAS have been designed, considering the key determinants of the patient's treatment decisions suggested by the evidence [41-45]. The pharmacological treatments such as the perception of efficacy, motivation, and general beliefs (3 items); association between drug dosage and tolerability (3 items); the importance of the speed of the effect on pain reduction (1 item); risk of physical dependence (1 item); modality of administration (3 items); side effects tolerability (1 item); the impact of the consumption on cognitive functioning (1 item) are evaluated in detail. On the contrary, the nonpharmacological treatments evaluated efficacy perception, motivation, and general beliefs (3 items); attitude toward interventions that act of mental representations and emotions behind the pain experience (1 item); attitude toward interventions mind-body (1 item); attitude toward interventions that might drive to immediate pain relief (1 item); attitude to short-term interventions (1 item) and that might be implemented autonomously and at-home (1 item); attitude toward interventions that self-administered (active role vs passive role) (1 item); attitude toward interventions that are conducted in a group versus separately (2 items).

In the second part, patients have to complete a decision tree. A decision tree has been defined as a sort of “abstract structure” able to increase knowledge about the choice that should be performed. The decision tree supports an informed and shared decision [46,47]. Through the decision tree, each patient will compare individual use associated with pharmacological and nonpharmacological treatments. In addition, each patient will identify each option (pharmacological vs nonpharmacological treatments), advantages, and disadvantages. Each of them will be assigned a value ranging from 1 to 10 (advantages-gains) and from −1 to −10 (disadvantages-losses). The total value obtained, given by the sum of the advantages and disadvantages for each option, permit to calculate the individual use associated with each treatment. Each patient is trained to fill the decision aid after 15 days from the discharge to the hospital, at 1 month from surgery, and at 3 months before the clinical follow-ups. The final aim of the decision aid is to support the construction and expression of patients’ preferences about current treatments for CP by comparing pharmacological versus nonpharmacological treatments.

Overall, the information collected by the PainRELife app through the self-reported measures and e-diary will be stored in the *Nu* platform and managed by the health care providers (oncologists, nurses, and psychologists) in order to monitor the health and psychological status of the patients and permitting the early identification of at-risk situations. Patients may see the results obtained in each self-reported measure and other tasks of the PainRELife app and are trained to contact the health care professionals for any questions about these issues. The results obtained in the assessment performed through PainRELife app will be discussed in the following clinical consultation.

The PainRELife app will be installed on the patient’s mobile phone, and if the patient does not have a personal mobile phone, it will be provided by the researcher (Multimedia Appendix 2). The PainRELife app can run on IOS and Android operating systems. To ensure timely communication with the hospitals and the physicians, relevant contact information will also be explicitly provided to the participants within the PainRELife app.

### Participants

In this study, 20 participants consecutively admitted at Istituto Europeo di Oncologia (IEO) at the Division of Medical Senology and the Division of Pain Therapy and Palliative Care with a diagnosis of breast cancer and CP will be enrolled. Such a sample size is recommended for 1 group [48,49]. Other studies suggested that in order to record a higher percentage of usability errors, it is advisable to enroll a sample of more than 12 participants [50]. Furthermore, within the PainRELife project, a mobile app was also configured for patients experiencing chronic pain post stroke and tested in a referral center for neurological rehabilitation. Thus, a parallel data collection will be conducted on a sample of 20 participants consecutively admitted at Casa di Cura Policlinico. The present protocol refers solely on IEO patients.

### Selection Criteria

Inclusion criteria include (1) patients >18 years; (2) having an early breast cancer diagnosis; (3) having undergone surgical intervention for breast carcinoma (quadrantectomy, mastectomy ± lymph node dissection); (4) the presence of postsurgical pain with a score ≥3 in the Numerical Rating Scale (NRS) or VAS; (5) patients with internet access and a personal smartphone; and (6) patients who have read and signed the informed consent.

Exclusion criteria include (1) the presence of primary psychiatric or neurological conditions preventing the ability to use the mobile app and the capacity to express free consent to join the study; (2) the presence of other medical conditions which imply an active antalgic treatment; and (3) patients who refused to sign the informed consent.

### Measures

#### Patient Demographic and Medical Variables

Age, gender, education, marital status, cancer diagnosis, type of surgery, oncological treatments, and comorbid medical disorders were collected through electronic medical records and self-reports.

#### Pain Self-Efficacy Questionnaire

The PSE is a self-administered questionnaire composed of 10 items rated on a 7-point Likert scale from “*not at all confident*” to “*completely confident.*” The Pain Self-Efficacy Questionnaire (PSEQ) [51,52] measures self-efficacy while performing activities of daily living, despite experiencing pain. The total score of the PSEQ ranges from 0 to 60. The PSEQ has been validated for Italian culture by Chiarotto et al [52] and showed high internal consistency (Cronbach α=.94).

#### Nine-Item Shared Decision-Making Questionnaire

The 9-item Shared Decision-Making Questionnaire (SDM-Q-9) [53] is a self-administered questionnaire composed of 9 items on a 6-point Likert scale from “*completely disagree*” to “*completely agree.*” The SDM-Q-9 evaluates patients’ perception of SDM and their level of involvement during the consultation, as well as the information received on possible treatments and potential risks and benefits of being involved in the decision-making process. The SDM-Q-9 showed high internal consistency in the original validation (Cronbach α=.94).

#### Mobile Application Rating Scale

The Mobile Application Rating Scale (MARS) [54] is a self-administered questionnaire specifically created to rate the quality of eHealth apps [55], and it is composed of 6 different sections (A, B, C, D, E, and F) for a total of 29 items with 5 possible answers. It assesses the quality of the app according to 4 specific dimensions: engagement experienced while using the app (A); functionality (B), aesthetics (C), quality of the information received (D), as well as the subjective perception of app quality (E) and the perceived impact on the app toward knowledge, attitudes, and probability to change user behaviors (F). The MARS showed a high internal consistency (Cronbach α=.90) and good psychometric properties [56].

#### VAS and NRS

The VAS and NRS [57] are unidimensional assessment scales for evaluating pain intensity. The first one graphically represents the amplitude of perceived pain through a 10-cm predesigned line. The left extremity corresponds to the “*absence of pain*,” whereas the right represents the ”*worst pain possible.*“ Next, the patient will be asked to draw a sign on the line representing the level of experienced pain. In the NRS scale, similarly, patients will be asked to verbally specify the number corresponding to their experienced pain, according to the following numerical graduation: (0: “*no pain*,” 1-3: “*mild pain*,” 4-6: “*moderate pain*,” 7-10: “*severe pain*”). The use of the VAS or NRS is determined by the patient’s degree of cognitive functioning.

### Recruitment and Follow-ups

Patients admitted to the Division of Medical Senology of the IEO will be enrolled by the palliative care physician and a trained psychologist from the Applied Division of Cognitive and Psychological Science. The patients enrolled will have an interview with the psychologist responsible for the data collection. The psychologist will install the PainRELife app and create a personal account. Each patient will use the app for 3 months, during which data extracted from the questionnaires will be sent to the *Nu* platform. Furthermore, at baseline (T0), each patient will fill a set of questionnaires (PSEQ, SDM-Q-9, and VAS or NRS), while at 3 months (T1), each patient will fill the same questionnaires and MARS.

The palliative care physician and the psychologists in charge of data collection will monitor the information transmitted from the PainRELife app to the *Nu* platform for the whole period of testing*.*

Patients will be informed that neither the platform nor the app will replace the clinical consultation. Furthermore, for any necessity, patients have to get in contact with the pertaining division.

A monthly telephonic interview will be provided to maintain motivation and monitor and detect any psychological, physical, and technical problems. In addition, this program includes 3 calls: 1 at the end of week 4, week 8, and week 12. Each call will last about 10 minutes and address concerns related to the use of the app or their involvement in the pilot study. Data collected through the PainRELife app will be sent to the *Nu* platform and consequently managed by the physician responsible for data collection.

### Statistical Considerations

All statistical analyses will be performed through SPSS (version 26; IBM Corp). Data analyzed will be referred to the primary end point, such as usability experience and app quality, which is assessed by the MARS questionnaire. The questionnaire delivers a mean quantitative score for each subscale (A, B, C, D, E, and F), with a mean score from 1 to 5 for the given answers and a total mean score of app quality and the subjective quality perceived by the patients. High mean scores (range 3-5) indicate a good qualitative level of app usability, both considering the global quality and the specific subdimensions [58]. Descriptive statistics of all the questionnaires included in the app will also be processed. Considering the secondary end points, the mean and SD of the PSEQ questionnaire scores will be calculated at T0 (baseline) and T1 (at 3 months) and compared using an appropriate statistic test. PSEQ mean scores will be interpreted according to the established normative reference values [52]. Furthermore, the number of accesses and usage time for each app section will be collected and analyzed. This additional analysis will provide insights into compliance (considering the total number of accesses required by the care pathway) and the perceived usefulness of the educational and decision aid sections.

According to the SDM-Q-9, the questionnaire's total raw score will be obtained by summing all the scale items, ranging from 0 to 45. Since it is more comprehensible, the raw score will be transformed into an interval scale from 0 to 100 (where 0 indicates the lowest possible level of SDM and 200 pinpoints the highest level of SDM) by multiplying the raw score by 20/9. Lastly, bivariate correlations among the questionnaires administered at T1 will be computed. Specifically, a positive correlation between the quality of the app scores (measured by MARS), the perceived self-efficacy despite the presence of pain (measured by PSEQ), and SDM scores (measured by SDM-Q-9) is expected.

### Ethics Approval

This pilot study follows the principles stated in the Declaration of Helsinki and subsequent amendments and the Good Clinical Practice Guideline. This protocol was assessed and certified by the ethical board of the European Institute of Oncology (approval number R1597/21-IEO 1701). Informed consent will be obtained from all participants. The informed consent will contain detailed information on the aims and the procedures of the study, as well as on the analysis plan, risks, and benefits for the participants. Participation in the study will be voluntary, and participants will not receive any compensation.

Furthermore, the study data are deidentified. Data will be stored in the IEO databases for 10 years. The study will comply with national and local data protection legislation. Further, all data will be anonymized. Data will be stored in the IEO databases for 10 years.

## Results

The recruitment process started in May 2022 and ended in October 2022.

## Discussion

### Principal Expected Findings

Considering the complexity of CP experience in the cancer domain, the international scientific community has advocated identifying cutting-edge approaches for CP management. A growing body of studies [8,10,20,31,58] has underlined that health technology solutions might be considered an encouraging and challenging opportunity to enhance patient-centered care and increase patient participation in the care pathway, SDM, and health-related quality of life. This pilot study protocol described here and testing patients' usability experience at 3 months of a digital and integrated ecosystem (PainRELife mobile app and *Nu* platform) for CP in a sample of patients with early breast cancer will permit to achieve of a series of key expected outcomes enriching results obtained from the previous studies.

The *first expected outcome* concerns the possibility of collecting a set of patient-based evidence about the usability experience of the PainRELife mobile app, which will permit to tune of the future version of this type of health technological solutions for patients with chronic pain. More specifically, the results about usability will permit to identify which features (in terms of functionality, aesthetics, and quality of the information provided) a mobile app for CP management should have to be used by patients to manage their care pathway and which are the patient’s beliefs about the perceived impact on health behaviors (eg, “*The mobile App will help me to improve health literacy about my pain permitting me to adopt behaviors able to reduce CP*”). A second expected outcome concerns the possibility of overcoming limitations highlighted by earlier studies about health technologies for the patient’s clinical management (eg, inadequate sample size or follow-ups) [20]. Indeed, using a pilot study design to evaluate usability experience will inform future studies evaluating effectiveness, thereby increasing the probability of achieving a better implementation of such integrated ecosystem into routine clinical practice and increasing user retention.

A third expected outcome concerns the patient’s home monitoring, contributing to reducing barriers to pain management related to poor medical resources, inadequate health system, and low health literacy [9]. In particular, we argue that the direct communication between the *Nu* platform (health care professionals’ perspective) and the PainRELife mobile app (patients’ perspective) will permit the ongoing tracking of outpatients' physical and psychological health status, allowing physicians to recognize alarm signals promptly. Furthermore, such an integrated ecosystem will permit monitoring clinical follow-ups, reducing the risk of patients' dropout.

Finally, a fourth expected outcome is implementing the decision aid to improve patients' ability to participate in their clinical decision, providing a better portrayal of patient SDM processes in pain management. In addition, we argue that the integration of a decision aid in *the* PainRELife mobile app will allow patients with breast cancer to deal with preference-sensitive decisions by clarifying their needs, values, and expected outcomes associated with the treatment for CP. Also, data collected through the decision aid and shared with the physician will permit us to understand the patient's perspective before the clinical consultation better. This better knowledge about patient perspective will facilitate patient and doctor interaction and consequently will support the achievement of a shared decision about CP care.

### Limitations

Despite the innovative clinical and methodological aspects of the current usability study protocol, some limitations should be considered. First, the patient’s usability experience of the PainRELife app is evaluated by MARS administration without measuring outcomes related to the care process and the clinical workflow. However, physicians’ perspectives and related clinical workflow have been considered in the design phase of the PainRELife *ecosystem,* both for developing the *Nu* platform (the interface used by the physician) and for the PainRELife app (the interface used by the patient)*.* As reported, the digitized care pathways (managed through the *Nu* platform and PainRELife app) were developed following a consensus procedure that included both the available guidelines and recommendations on CP management and the local experience at the referral centers involved in the project (IEO and Casa di Cura Policlinico). This could have contained the risk of implementation bias. Second, a monthly telephonic interview is performed by a trained researcher for the monitoring of usage and concerns to maintain patient involvement in the study, reducing the risk of dropout and increasing the quality of the data collected. Notwithstanding, we have to consider the risk that monthly telephonic interviews are the outcome measures. Indeed, patients might have increased the usage of the PainRELife app to satisfy research expectations (*social desirability effect*) and because the researcher has dedicated attention to the participant (*Hawthorne effect*).

Finally, the last limitation concerns the decision to use a single-group instead a 2-group design for evaluating usability experience according to Julious [49] guidelines for a pilot study. A study design with 2 groups (intervention vs control group) could have also permitted an evaluation of differences in pain management associated with using the PainRELife app. This type of information could have informed a future effectiveness study.

### Conclusions

We expect to provide a set of suggestions for the development and implementation of this health technology into routine clinical practice. Data from this pilot study will inform the conduction of full-powered studies testing the efficacy and impact of digital support systems for CP management. Further, the future implementation of this integrated ecosystem will improve CP management in patients with cancer and SDM models through decision aid.

## Appendix

Multimedia Appendix 1 PainRELife Decision Aid.

Multimedia Appendix 2 Screenshots taken on an iPhone device illustrating the patient interface of the mobile application.

## Abbreviations

CP: chronic pain
FHIR: Fast Healthcare Interoperability Resources
IEO: Istituto Europeo di Oncologia
MARS: Mobile Application Rating Scale
NRS: Numerical Rating Scale
PSEQ: Pain Self-Efficacy Questionnaire
SDM-Q-9: Shared Decision-Making Questionnaire 9-Item
SDM: shared decision-making
VAS: Visual Analogue Scale

## Data Availability

The data sets generated and analyzed during the current study are available from the corresponding author upon reasonable request.

## References

[1] Hamood R, Hamood H, Merhasin I, Keinan-Boker L (2018). Chronic pain and other symptoms among breast cancer survivors: prevalence, predictors, and effects on quality of life. Breast Cancer Res Treat.

[2] Lam E, Wong G, Zhang L, Drost L, Karam I, Yee C, McCurdy-Franks E, Razvi Y, Ariello K, Wan BA, Nolen A, Wang K, DeAngelis C, Chow E (2021). Self-reported pain in breast cancer patients receiving adjuvant radiotherapy. Support Care Cancer.

[3] Leysen L, Adriaenssens N, Nijs J, Pas R, Bilterys T, Vermeir S, Lahousse A, Beckwée D (2019). Chronic pain in breast cancer survivors: nociceptive, neuropathic, or central sensitization pain?. Pain Pract.

[4] Bao T, Seidman A, Li Q, Seluzicki C, Blinder V, Meghani SH, Farrar JT, Mao JJ (2018). Living with chronic pain: perceptions of breast cancer survivors. Breast Cancer Res Treat.

[5] Smith SK, MacDermott K, Amarasekara S, Pan W, Mayer D, Hockenberry M (2019). Reimagine: a randomized controlled trial of an online, symptom self-management curriculum among breast cancer survivors. Support Care Cancer.

[6] Duenas M, Ojeda B, Salazar A, Mico JA, Failde I (2016). A review of chronic pain impact on patients, their social environment and the health care system. J Pain Res.

[7] Cox-Martin E, Anderson-Mellies A, Borges V, Bradley C (2020). Chronic pain, health-related quality of life, and employment in working-age cancer survivors. J Cancer Surviv.

[8] Zheng C, Chen X, Weng L, Guo L, Xu H, Lin M, Xue Y, Lin X, Yang A, Yu L, Xue Z, Yang J (2020). Benefits of mobile apps for cancer pain management: systematic review. JMIR Mhealth Uhealth.

[9] Dorfman CS, Kelleher SA, Winger JG, Shelby RA, Thorn BE, Sutton LM, Keefe FJ, Gandhi V, Manohar P, Somers TJ (2018). Development and pilot testing of an mHealth behavioral cancer pain protocol for medically underserved communities. J Psychosoc Oncol.

[10] Lalloo C, Shah U, Birnie KA, Davies-Chalmers C, Rivera J, Stinson J, Campbell F (2017). Commercially available smartphone apps to support postoperative pain self-management: scoping review. JMIR Mhealth Uhealth.

[11] Kondylakis H, Bucur A, Dong F, Renzi C, Manfrinati A (2017). iManageCancer: developing a platform for empowering patients and strengthening self-management in cancer diseases.

[12] Marceglia S, Conti C (2017). A technology ecosystem for chronic pain: promises, challenges, and future research. Mhealth.

[13] Hernandez Silva E, Lawler S, Langbecker D (2019). The effectiveness of mHealth for self-management in improving pain, psychological distress, fatigue, and sleep in cancer survivors: a systematic review. J Cancer Surviv.

[14] Kapoor A, Nambisan P, Baker E (2020). Mobile applications for breast cancer survivorship and self-management: a systematic review. Health Informatics J.

[15] Sundararaman LV, Edwards RR, Ross EL, Jamison RN (2017). Integration of mobile health technology in the treatment of chronic pain. Reg Anesth Pain Med.

[16] Briggs LG, Labban M, Alkhatib K, Nguyen DD, Cole AP, Trinh QD (2022). Digital technologies in cancer care: a review from the clinician's perspective. J Comp Eff Res.

[17] Stone AA, Broderick JE (2007). Real-time data collection for pain: appraisal and current status. Pain Med.

[18] Heron KE, Smyth JM (2010). Ecological momentary interventions: incorporating mobile technology into psychosocial and health behaviour treatments. Br J Health Psychol.

[19] Rost S, Van Ryckeghem DML, Koval P, Sütterlin S, Vögele C, Crombez G (2016). Affective instability in patients with chronic pain: a diary approach. Pain.

[20] Penedo FJ, Oswald LB, Kronenfeld JP, Garcia SF, Cella D, Yanez B (2020). The increasing value of eHealth in the delivery of patient-centred cancer care. Lancet Oncology.

[21] Paice JA, Portenoy R, Lacchetti C, Campbell T, Cheville A, Citron M, Constine LS, Cooper A, Glare P, Keefe F, Koyyalagunta L, Levy M, Miaskowski C, Otis-Green S, Sloan P, Bruera E (2016). Management of chronic pain in survivors of adult cancers: American Society of Clinical Oncology clinical practice guideline. J Clin Oncol.

[22] Satija A, Ahmed SM, Gupta R, Ahmed A, Rana SPS, Singh SP, Mishra S, Bhatnagar S (2014). Indian J Med Res.

[23] Gorini A, Riva S, Marzorati C, Cropley M, Pravettoni G (2018). Rumination in breast and lung cancer patients: preliminary data within an Italian sample. Psychooncology.

[24] Stacey D, Légaré F, Lewis K, Barry MJ, Bennett CL, Eden KB, Holmes-Rovner M, Llewellyn-Thomas H, Lyddiatt A, Thomson R, Trevena L (2017). Decision aids for people facing health treatment or screening decisions. Cochrane Database Syst Rev.

[25] Elwyn G, O'Connor A, Stacey D, Volk R, Edwards A, Coulter A, Thomson R, Barratt A, Barry M, Bernstein S, Butow P, Clarke A, Entwistle V, Feldman-Stewart D, Holmes-Rovner M, Llewellyn-Thomas H, Moumjid N, Mulley A, Ruland C, Sepucha K, Sykes A, Whelan T, International Patient Decision Aids Standards (IPDAS) Collaboration (2006). Developing a quality criteria framework for patient decision aids: online international Delphi consensus process. BMJ.

[26] Covvey JR, Kamal KM, Gorse EE, Mehta Z, Dhumal T, Heidari E, Rao D, Zacker C (2019). Barriers and facilitators to shared decision-making in oncology: a systematic review of the literature. Support Care Cancer.

[27] Col N, Hull S, Springmann V, Ngo L, Merritt E, Gold S, Sprintz M, Genova N, Nesin N, Tierman B, Sanfilippo F, Entel R, Pbert L (2020). Improving patient-provider communication about chronic pain: development and feasibility testing of a shared decision-making tool. BMC Med Inform Decis Mak.

[28] Omaki E, Castillo R, McDonald E, Eden K, Davis S, Frattaroli S, Rothman R, Shields W, Gielen A, My Healthy Choices Decision Aid Team (2021). A patient decision aid for prescribing pain medication: results from a pilot test in two emergency departments. Patient Educ Couns.

[29] Yung A, Kay J, Beale P, Gibson KA, Shaw T (2021). Computer-based decision tools for shared therapeutic decision-making in oncology: systematic review. JMIR Cancer.

[30] Munzone E, Bagnardi V, Campennì G, Mazzocco K, Pagan E, Tramacere A, Masiero M, Iorfida M, Mazza M, Montagna E, Cancello G, Bianco N, Palazzo A, Cardillo A, Dellapasqua S, Sangalli C, Pettini G, Pravettoni G, Colleoni M, Veronesi P (2019). Preventing chemotherapy-induced alopecia: a prospective clinical trial on the efficacy and safety of a scalp-cooling system in early breast cancer patients treated with anthracyclines. Br J Cancer.

[31] Day FC, Pourhomayoun M, Keeves D, Lees AF, Sarrafzadeh M, Bell D, Pfeffer MA (2019). Feasibility study of an EHR-integrated mobile shared decision making application. Int J Med Inform.

[32] Masiero M, Cutica I, Russo S, Mazzocco K, Pravettoni G (2018). Psycho-cognitive predictors of burnout in healthcare professionals working in emergency departments. J Clin Nurs.

[33] Oliveri S, Renzi C, Masiero M, Pravettoni G (2015). Living at risk: factors that affect the experience of direct-to-consumer genetic testing. Mayo Clin Proc.

[34] Shah U, Chiew T (2019). A systematic literature review of the design approach and usability evaluation of the pain management mobile applications. Symmetry.

[35] Nayebi F, Desharnais J, Abran A (2012). The state of the art of mobile application usability evaluation.

[36] Filipponi C, Masiero M, Pizzoli SFM, Grasso R, Ferrucci R, Pravettoni G (2022). A comprehensive analysis of the cancer chronic pain experience: a narrative review. Cancer Manag Res.

[37] Zigmond AS, Snaith RP (1983). The hospital anxiety and depression scale. Acta Psychiatr Scand.

[38] Sullivan MJL, Bishop SR, Pivik J (1995). The pain catastrophizing scale: development and validation. Psychol Assess.

[39] Cleeland CS, Ryan KM (1994). Pain assessment: global use of the brief pain inventory. Ann Acad Med Singapore.

[40] Caraceni A, Mendoza TR, Mencaglia E, Baratella C, Edwards K, Forjaz MJ, Martini C, Serlin RC, de Conno F, Cleeland CS (1996). A validation study of an Italian version of the Brief pain inventory (Breve Questionario per la Valutazione del Dolore). Pain.

[41] Becker WC, Dorflinger L, Edmond SN, Islam L, Heapy AA, Fraenkel L (2017). Barriers and facilitators to use of non-pharmacological treatments in chronic pain. BMC Fam Pract.

[42] Mühlbacher AC, Kaczynski A, Zweifel P, Johnson FR (2016). Experimental measurement of preferences in health and healthcare using best-worst scaling: an overview. Health Econ Rev.

[43] Müller-Schwefe GHH, Wimmer AM, Dejonckheere J, Eggers A, Vellucci R (2014). Patients' and physicians' perspectives on opioid therapy for chronic cancer and musculoskeletal pain in Germany, Italy, and Turkey: PAin RESearch (PARES) survey. Curr Med Res Opin.

[44] Kandemir D, Oskay Ü (2017). Sexual problems of patients with urostomy: a qualitative study. Sex Disabil.

[45] Turk D, Boeri M, Abraham L, Atkinson J, Bushmakin A, Cappelleri J, Hauber B, Klein K, Russo L, Viktrup L, Walsh D (2020). Patient preferences for osteoarthritis pain and chronic low back pain treatments in the United States: a discrete-choice experiment. Osteoarthritis Cartilage.

[46] Gheondea-Eladi A (2019). Patient decision aids: a content analysis based on a decision tree structure. BMC Med Inform Decis Mak.

[47] Gorini A, Masiero M, Pravettoni G (2016). Patient decision aids for prevention and treatment of cancer diseases: are they really personalised tools?. Eur J Cancer Care (Engl).

[48] NCSS (2018). Pilot study sample size rules of thumb. PASS Sample Size Software.

[49] Julious SA (2005). Sample size of 12 per group rule of thumb for a pilot study. Pharm Stat.

[50] Schmettow M (2012). Sample size in usability studies. Commun ACM.

[51] Nicholas MK (1989). Self-efficacy and chronic pain.

[52] Chiarotto A, Vanti C, Ostelo RW, Ferrari S, Tedesco G, Rocca B, Pillastrini P, Monticone M (2015). The pain self-efficacy questionnaire: cross-cultural adaptation into Italian and assessment of its measurement properties. Pain Pract.

[53] Goss C, Ghilardi A, Deledda G, Buizza C, Bottacini A, Del Piccolo L, Rimondini M, Chiodera F, Mazzi MA, Ballarin M, Bighelli I, Strepparava MG, Molino A, Fiorio E, Nortilli R, Caliolo C, Zuliani S, Auriemma A, Maspero F, Simoncini EL, Ragni F, Brown R, Zimmermann C (2013). INvolvement of breast CAncer patients during oncological consultations: a multicentre randomised controlled trial--the INCA study protocol. BMJ Open.

[54] Domnich A, Arata L, Amicizia D, Signori A, Patrick B, Stoyanov S, Hides L, Gasparini R, Panatto D (2016). Development and validation of the Italian version of the mobile application rating scale and its generalisability to apps targeting primary prevention. BMC Med Inform Decis Mak.

[55] Stoyanov SR, Hides L, Kavanagh DJ, Zelenko O, Tjondronegoro D, Mani M (2015). Mobile app rating scale: a new tool for assessing the quality of health mobile apps. JMIR Mhealth Uhealth.

[56] Terhorst Y, Philippi P, Sander LB, Schultchen D, Paganini S, Bardus M, Santo K, Knitza J, Machado GC, Schoeppe S, Bauereiß N, Portenhauser A, Domhardt M, Walter B, Krusche M, Baumeister H, Messner E (2020). Validation of the mobile application rating scale (MARS). PLoS One.

[57] Jensen MP, McFarland CA (1993). Increasing the reliability and validity of pain intensity measurement in chronic pain patients. Pain.

[58] Salazar A, de Sola H, Failde I, Moral-Munoz JA (2018). Measuring the quality of mobile apps for the management of pain: systematic search and evaluation using the mobile app rating scale. JMIR Mhealth Uhealth.

