# Supplementary figures and images for: Usability Testing of a New Digital Integrated Health Ecosystem (PainRELife) for the Clinical Management of Chronic Pain in Patients With Early Breast Cancer: Protocol for a Pilot Study
Source: JMIR Res Protoc. 2023 May 12;12:e41216. doi: 10.2196/41216 (PMC10221507; doi:10.2196/41216)

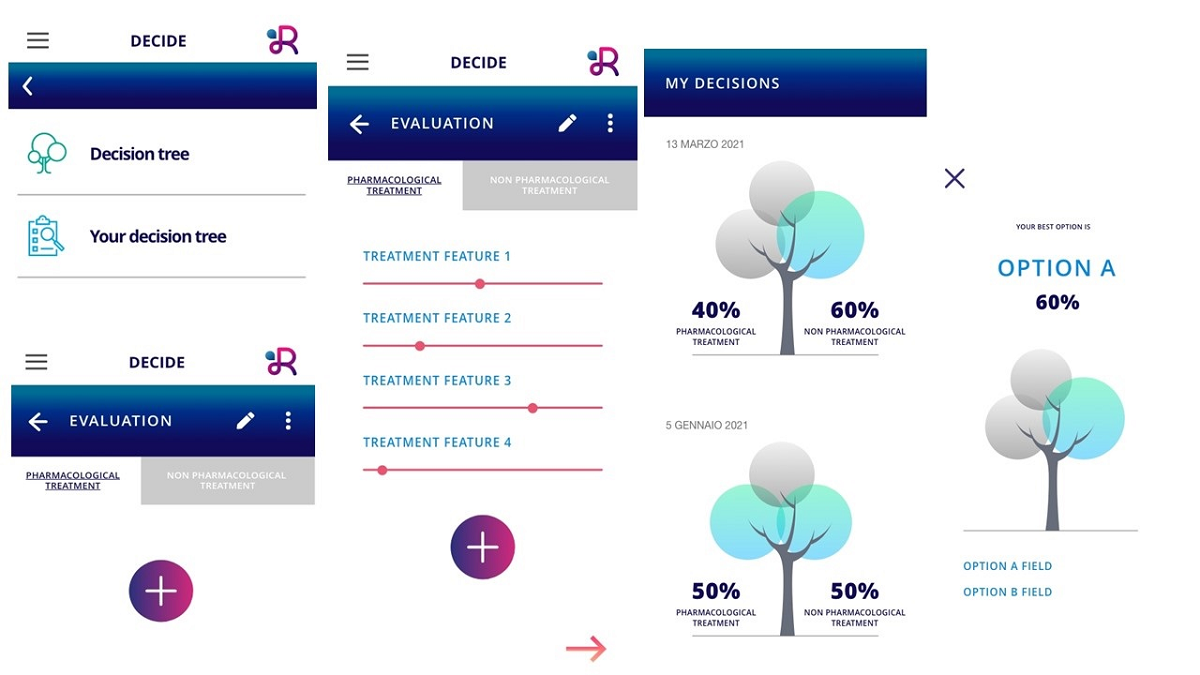

Supplement: Multimedia Appendix 1 [file resprot_v12i1e41216_app1.png]

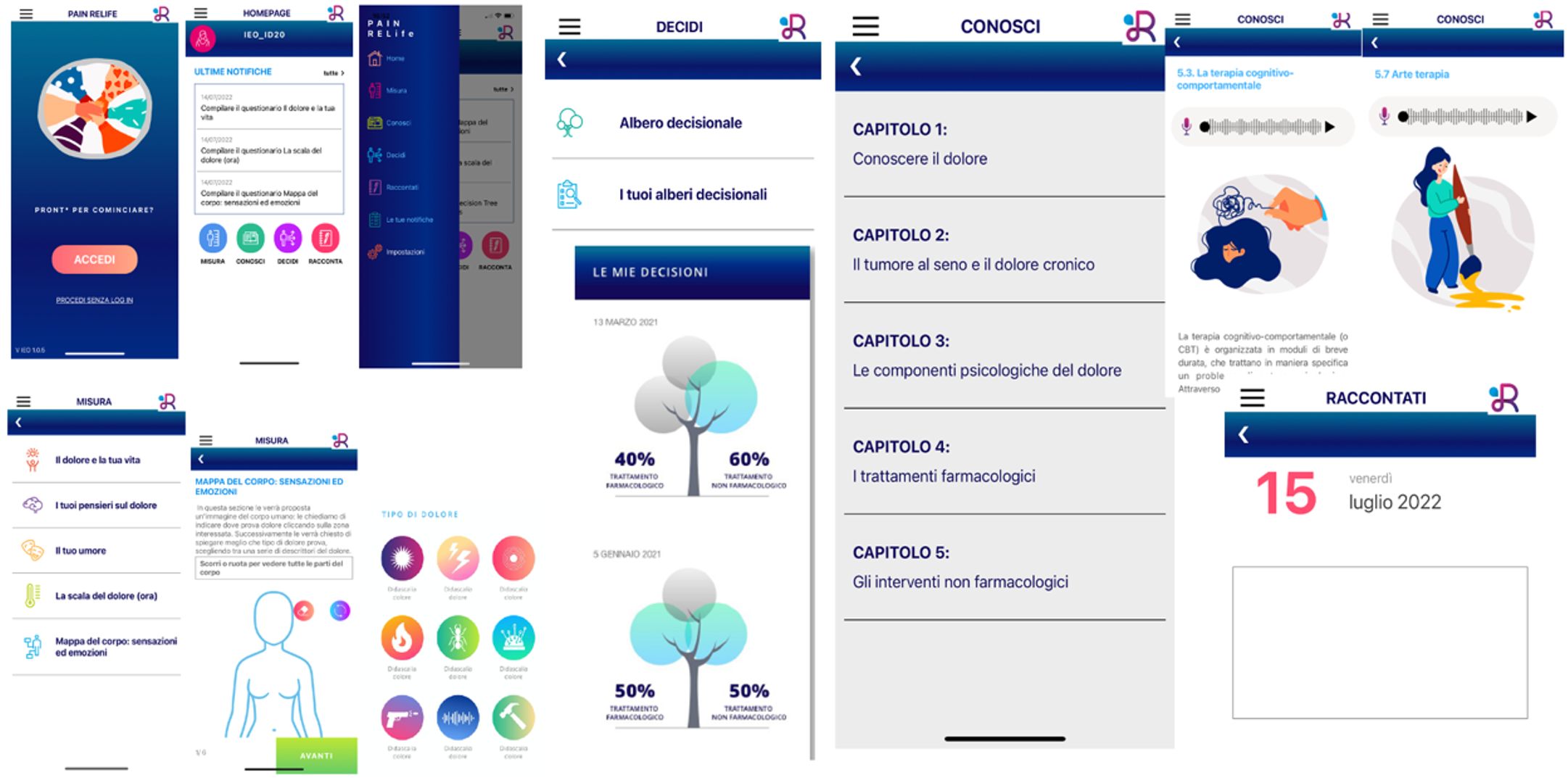

Supplement: Multimedia Appendix 2 [file resprot_v12i1e41216_app2.png]
